# Integrative bioinformatics analysis characterizing the role of *EDC3* in mRNA decay and its association to intellectual disability

**DOI:** 10.1186/s12920-018-0358-6

**Published:** 2018-04-23

**Authors:** Ute Scheller, Kathrin Pfisterer, Steffen Uebe, Arif B. Ekici, André Reis, Rami Jamra, Fulvia Ferrazzi

**Affiliations:** 1 Institute of Human Genetics, Friedrich-Alexander-Universität Erlangen-Nürnberg, Schwabachanlage 10, 91054 Erlangen, Germany; 2 Institute of Human Genetics, University of Leipzig, Philipp-Rosenthal-Straße 55, 04103 Leipzig, Germany

**Keywords:** EDC3, mRNA degradation, Intellectual disability, Transcriptome analysis, Pathways, Co-expression network

## Abstract

**Background:**

Decapping of mRNA is an important step in the regulation of mRNA turnover and therefore of gene expression, which is a key process controlling development and homeostasis of all organisms. It has been shown that EDC3 plays a role in mRNA decapping, however its function is not well understood. Previously, we have associated a homozygous variant in *EDC3* with autosomal recessive intellectual disability. Here, we investigate the functional role of EDC3.

**Methods:**

We performed transcriptome analyses in patients’ samples. In addition, we established an EDC3 loss-of-function model using siRNA-based knockdown in the human neuroblastoma cell line SKNBE and carried out RNA sequencing. Integrative bioinformatics analyses were performed to identify EDC3-dependent candidate genes and/or pathways.

**Results:**

Our analyses revealed that 235 genes were differentially expressed in patients versus controls. In addition, AU-rich element (ARE)-containing mRNAs, whose degradation in humans has been suggested to involve EDC3, had higher fold changes than non-ARE-containing genes. The analysis of RNA sequencing data from the EDC3 in vitro loss-of-function model confirmed the higher fold changes of ARE-containing mRNAs compared to non-ARE-containing mRNAs and further showed an upregulation of long non-coding and coding RNAs. In total, 764 genes were differentially expressed. Integrative bioinformatics analyses of these genes identified dysregulated candidate pathways, including pathways related to synapses/coated vesicles and DNA replication/cell cycle.

**Conclusion:**

Our data support the involvement of EDC3 in mRNA decay, including ARE-containing mRNAs, and suggest that EDC3 might be preferentially involved in the degradation of long coding and non-coding RNAs. Furthermore, our results associate ECD3 loss-of-function with synapses-related pathways. Collectively, our data provide novel information that might help elucidate the molecular mechanisms underlying the association of intellectual disability with the dysregulation of mRNA degradation.

**Electronic supplementary material:**

The online version of this article (10.1186/s12920-018-0358-6) contains supplementary material, which is available to authorized users.

## Background

Gene expression can be regulated at different points during the processing of genetic information, including transcription, mRNA processing, translation, and degradation [1]. Previously, several RNA decay pathways have been identified [2–4]. The two major exonucleolytic mRNA decay pathways, the 5′ to 3′ and the 3′ to 5’ mRNA decay, both start with the deadenylation of the 3′ end poly(A) tail [3, 4]. In the 5′ to 3’ mRNA degradation pathway a major player is the human decapping factor 2 (DCP2), which hydrolyses the 7-methyl-guanosine cap at the 5′ end directly after the deadenylation of the poly(A) tail [5, 6]. The decapped mRNA is degraded by the 5′ to 3′ exonuclease Xrn1 [4]. In recent years, it has become clear that a number of different decapping complexes exist in order to enable transcript specificity [7]. How this transcript specificity is achieved in humans and how defects in its regulatory pathways affect human health remains mainly elusive. Multiple co-factors have been identified that enhance the decapping activity of DCP2 [8], among them the enhancer of decapping 3 (EDC3), containing the three domains LSm, FDF, and YjeF-N [9, 10]. In humans EDC3 has been found to interact with the RNA binding protein Tristetraprolin (TTP, known as well as ZFP36 [11]), which binds to AU-rich elements (AREs) in mRNAs and enhances the decapping and degradation of ARE-containing mRNAs [9]. Importantly, in a previous study, we identified a homozygous variant in *EDC3* (c.161T>C; p.Phe54Ser) in two children of a consanguineous family affected by mild non-syndromic intellectual disability [12], indicating that EDC3 and DCP2 contribute to neuronal functions. Molecular modeling predicted the identified variant to significantly disrupt the hydrophobic LSm domain of EDC3 [12], which has been shown to be important for the interaction between Edc3 and Dcp2 in yeast [13–15]. Additionally, functional analyses showed that the altered EDC3 was unable to enhance the decapping activity of DCP2 at low concentrations and inhibited DCP2 activity at high concentrations [12]. However, the genome wide transcriptional effect of *EDC3* impairment in human cells has not yet been investigated. Transcriptome analysis has previously been successfully used to help unravel the functional consequences of identified variants in intellectual disability [16–18]. Therefore, we conducted RNA sequencing (RNA-seq) on lymphoblastoid cell lines from the two patients as well as samples from an EDC3 loss-of-function model in the neuroblastoma cell line SKNBE. The analysis of our RNA-seq data revealed RNA classes that appear to be preferentially affected by EDC3 loss-of-function and identified dysregulated candidate pathways. Thus, our data contribute to the understanding of the pathomechanism of intellectual disability in our patients and in general.

## Methods

### Cells and cell culture

Lymphocytes were extracted from patients’ blood and transformed with Epstein-Barr virus to establish a lymphoblastoid cell line. Cells were kept as suspension in RPMI-1640 (Biochrom), 20% fetal calf serum (FCS), 1% Penicillin/ Streptomycin and 2 mM L-Glutamine (Gibco) at 37 °C, 5% CO_2_ and 91% humidity.

The human neuroblastoma cell line SKNBE was cultivated in Dulbecco’s Modified Eagle’s Medium (DMEM/HAM’s F12) complemented with 10% FCS, 1% Penicillin/Streptomycin and 0.5 mmol/l L-Glutamine (Gibco). Cells were cultured at 37 °C, 5% CO_2_ and 91% humidity.

### Transfection, RNA isolation, cDNA synthesis and quantitative real-time PCR

The neuroblastoma cell line SKNBE was transfected with siRNAs to knock down gene expression of *EDC3*. Three different Silencer Select Pre-designed siRNAs targeting EDC3 (hereafter indicated as siEDC3-1, siEDC3-2, siEDC3-3; Invitrogen) (Additional file 1: Table S1) were utilized at a 10 nM concentration. Scrambled siRNA and siRNA targeting GAPDH (Invitrogen) were used as negative and positive control, respectively. Lipofectamine® RNAiMAX (Invitrogen) was used as transfection agent. 24 h prior to the siRNA knockdown, 150,000 SKNBE cells per well were seated in a six well plate in antibiotic-free proliferation medium. For cell differentiation, 10 μM retinoic acid and 25 μM caffeine acid were added to the proliferation medium during transfection, as in Redova et al. [19]. In parallel to the differentiation, not transfected cells were treated with normal proliferation medium (DMEM/HAM’s F12 + 10% FCS).

Total RNA was extracted 72 h after transfection using RNeasy Mini Kit and Qiashredder Kit (Qiagen) according to the manufacturer’s protocol. Following standard protocols (Invitrogen), Superscript II and Random Primers (Invitrogen) were used to transcribe RNA into cDNA. Real Time PCR was employed to analyze the gene expression level of *EDC3* in transfected cells and validate the transfection result. Predesigned TaqMan probes (EDC3: Hs00257810_m1) and TaqMan Gene Expression Mastermix (Applied Biosystems) were used according to manufacturer’s protocol and assays were run on QuantStudio 12 K Flex real-time PCR System (Applied Biosystems). The gene expression of *EDC3* was normalized against the average of the four endogenous controls ß-Actin (*huACTB*), ß-2-microglobulin (*huB2M*), acidic ribosomal protein (*huPO*), and transcription-factor IID (*huTBP*) (Applied Biosystems). Four technical replicates were performed for each assay.

RNA from lymphoblastoid cell line samples was extracted and transcribed into cDNA following the same procedure as for SKNBE.

### RNA sequencing

For RNA-seq analysis of patients’ samples, libraries were prepared from the RNA extracted from lymphoblastoid cell lines using Nugen Ovation Human FFPE RNA-seq Kit according to the manufacturer’s instructions (Nugen Technologies, San Carlos, CA) and subjected to single-end sequencing on a SOLiD 4 platform (Life Technologies, Carlsbad, CA). LifeScope analysis suite was employed to align reads to the hg19 reference genome. Subread’s featureCounts v.1.4.6 [20] was used to produce absolute read counts per gene using Ensembl’s gtf annotation file (genebuild 2013-09) for hg19.

For RNA-seq analysis of SKNBE samples, barcoded RNA sequencing libraries were prepared as previously described [21] and sequenced on a HighSeq-2500 platform (Illumina, San Diego, CA). After mapping of the single-end reads [21], absolute read counts per gene were produced using Subread’s featureCounts and the Ensembl’s gtf annotation file. Transcriptome data were validated by quantitative PCR (Additional file 2: Figure S1).

### Differential expression analysis

All analyses were performed using R version 3.3.0 [22]. Differential expression analysis was performed with the DESeq2 package v.1.12.3 [23]. Genes were declared differentially expressed (DEGs) if their Benjamini-adjusted *p*-value was lower than 0.1. Only expressed genes (i.e. passing the independent filtering performed by DESeq2) were used for all following analyses.

In order to identify genes coding for ARE-containing RNAs, the AU-rich element-containing mRNA database (ARED) was utilized [24] to classify genes as ARE-containing and non-ARE-containing.

The allocation of genes to different functional classes, such as protein coding, antisense, lincRNA, was based on biotypes from the Ensembl’s gtf annotation file. HGNC gene symbols, where available, were assigned to Ensembl genes relying on biomaRt package v.2.28.0. For each gene the length provided by featureCounts was used, which corresponds to the length of the union of all exons of the gene. Long non-coding genes were taken as those classified to biotypes: lincRNA, antisense, processed_transcript, sense_intronic, sense_overlapping, 3prime_overlapping_ncrna. In order to analyze the behavior of protein coding genes with different lengths, genes were sorted by increasing length and binned into deciles.

In order to evaluate the expression of SKNBE DEGs in different tissues, normalized expression data (FPKM) of “Illumina Body Map” were retrieved from Gene Expression Atlas [25]. These consist of expression values for 33,413 genes from 16 different tissues contained in highly curated RNA-seq and microarray experiments from ArrayExpress [26]. Tissue expression profiles of DEGs with an associated HGNC symbol could be extracted from the Atlas and clustered using hierarchical clustering (hclust function in R/Bioconductor).

For analyses on a protein level a human protein-protein interaction network (PPI) published by Li et al. [27] was employed. This network was based on interactions contained in the BioGrid database [28]; pre-processing done by the authors included removing isolated nodes, self-interacting edges and human-non-human interacting proteins. After pre-processing, the authors clustered the network nodes into 816 modules based on dense interactions within the modules and only sparse interactions between them. The DEGs in SKNBE were mapped onto this network and enriched modules were identified by means of the hypergeometric test.

All functional annotation analyses were performed using DAVID v. 6.7 [29, 30], employing Gene Ontology (GO_FAT), KEGG and UniProt tissue expression (UP_TISSUE) as annotations.

### Module detection using weighted gene co-expression network analysis

Weighted gene co-expression network analysis (WGCNA) of SKNBE DEGs was performed using the R/Bioconductor package WGCNA version 1.51 [31]. First, a signed co-expression similarity matrix was constructed, which describes pairwise similarities of gene expression profiles relying on Pearson’s correlation. Next, the weighted adjacency matrix was built by raising the similarity matrix values to a soft-thresholding power, which in this study was taken equal to β = 30.

The weighted co-expression network was employed to identify modules, i.e. clusters of highly interconnected genes and thus with highly correlated expression profiles across samples. Module detection in the package relies on the calculation of a topological overlap measure (TOM) dissimilarity matrix, which serves as input for average linkage hierarchical clustering. Branches from the resulting tree were divided into modules using the DynamicTreeCut Algorithm with the option deepSplit = 0. The choice of the value that specifies the sensitivity of splitting clusters was guided by visual inspection of the TOM plot, a color-coded depiction of the dissimilarity matrix values. In our study the value was taken equal to 0 in order to obtain a small amount of modules, each containing genes with highly similar expression profiles. As measurement of how well a gene belongs to its module, a module membership (MM) score was calculated: this is defined as the correlation between a gene and the module’s eigengene, which can be thought of as the representative meta-gene for its module. Color-coded representations of expression profiles in each module were obtained with the R/Bioconductor package LSD v. 3.0 (https://CRAN.R-project.org/package=LSD).

## Results

### Transcriptome analysis of patients with *EDC3* variant

To explore the effects of the identified *EDC3* variant (c.161T>C; p.Phe54Ser) on gene expression, we performed RNA-seq of RNA extracted from lymphoblastoid cell lines of the two patients with the *EDC3* variant and two controls (Additional file 3: Table S2). Out of the 22,123 expressed genes, 235 were identified as differentially expressed (DEGs) (Benjamini adjusted *p*-value < 0.1; Additional file 4: Table S3). Functional enrichment analysis of DEGs performed with DAVID [29, 30] showed that the top enriched pathways are related to plasma membrane (Additional file 5: Table S4).

### *EDC3* variant is associated with differential expression of ARE-containing mRNAs

**Fig. 1:**
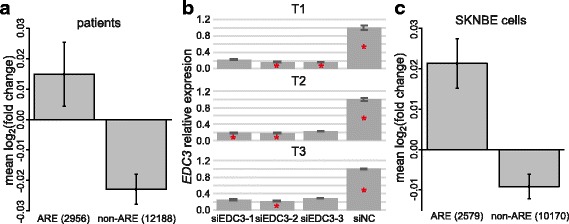
EDC3 loss-of-function is associated with differential expression of ARE-containing RNAs. **a** Differential expression of genes coding for ARE-containing RNAs in patients’ lymphoblastoid cell line samples**.** Out of the 22,123 expressed genes, 15,144 were listed in the database and 2956 of them were classified as ARE-containing. Bars indicate the standard deviation of the log_2_(fold change). **b** Expression of *EDC3* measured by real-time PCR after transfecting SKNBE cells with three different siRNAs targeting *EDC3* (siEDC3-1, siEDC3-2, siEDC3-3) as well as a negative control (siNC). In siNC samples, *EDC3* expression is set to 1. Gene expression levels are calculated from CT values of each sample, normalized against the mean CT value of the endogenous controls *ACTB*, *B2M*, *PO*, and *TBP*. T1-T3 refer to three biological replicate experiments. Samples chosen for RNA-seq are marked with a red asterisk. Bars indicate the standard deviation calculated on four technical replicates performed for each assay. **c** Differential expression of genes coding for ARE-containing RNAs in SKNBE cell line samples. Out of the 16,321 expressed genes, 12,749 were listed in the ARE-database and 2579 of them were classified as ARE-containing. Bars indicate the standard deviation of the log_2_(fold change)

As EDC3 has been found to interact with TTP and TTP enhances the decapping of ARE-containing mRNAs [9], we hypothesized that, if EDC3 function is impaired, expression of ARE-containing mRNAs is increased. Thus, we utilized the information contained in the ARE-database [24] to specifically assess the expression of ARE-containing RNAs in the transcriptome data obtained from patients’ and controls’ lymphoblastoid cell lines. Our results showed that the mean fold change of genes coding for ARE-containing RNAs was significantly higher than that of genes coding for non-ARE-containing RNAs (*p* = 5.93*10^− 4^; one-sided Student’s t-test; Fig. 1a). These data strengthen the hypothesis that the *EDC3* variant (c.161T>C; p.Phe54Ser) impairs mRNA decapping and might be causative of the patients’ phenotype.

### *EDC3* knockdown in SKNBE affects the expression of ARE-containing RNAs

Mechanisms exist to confer transcript specificity in mRNA degradation [7]. Thus, the analysis of lymphoblastoid cell lines might not provide an explanation of the observed intellectual disability of the two patients with the *EDC3* variant (c.161T>C; p.Phe54Ser). As RNA-seq analysis on neuronal patients’ cells cannot be performed, we established an EDC3 loss-of-function model in the neuroblastoma cell line SKNBE. To this aim, we knocked down *EDC3* using three different siRNAs (hereafter named siEDC3-1, siEDC3-2, siEDC3-3) during neuronal differentiation and performed three biological replicate experiments (indicated as T1-T3). The knockdown was successful and the expression of *EDC3* was reduced to less than 25% in most experiments (Fig. 1b). This knockdown did not affect the gross morphological differentiation of SKNBE cells after 72 h and after 5 days (Additional file 6: Figure S2).

RNA sequencing was performed for five knockdown and three control samples (Additional file 7: Table S5). The knockdown samples comprise the three siEDC3-2 samples from the three biological replicates T1-T3 and one sample each for siEDC3-1 and siEDC3-3 (respectively from T2 and T1, in which these siRNAs achieved the best knockdown). A principal component analysis plot of the 16,321 expressed genes shows that the expression profiles for the three controls cluster together and analogously those of the three replicates of siEDC3-2 knockdown; in addition, expression profiles associated with all knockdowns are clearly separated from controls (Additional file 8: Figure S3).

As in patients’ lymphoblastoid cell lines with the *EDC3* variant, also the neuronal cell line SKNBE upon *EDC3* knockdown showed in the transcriptome analysis that ARE-containing genes have a higher mean fold change than non-ARE-containing genes (*p* = 5.02*10^− 6^; one-sided Student’s t-test) (Fig. 1c). This concurrence is further evidence of the pathogenicity of the reported variant in our two patients in the sense of a loss-of-function variant [12].

### *EDC3* knockdown in SKNBE is associated with upregulation of long-noncoding and long coding RNAs

**Fig. 2:**
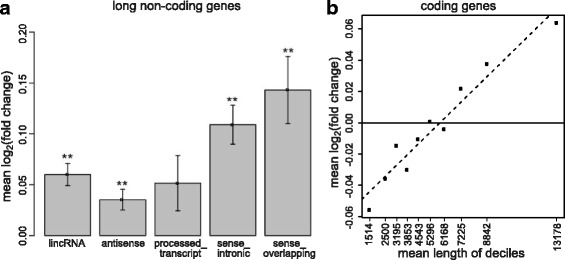
Differential expression of long non-coding and coding RNAs. **a** Mean log_2_(fold change) of long non-coding genes in different categories; bars indicate the standard deviation of log_2_(fold change); **: *p*-value < 0.001 (one-sided Student’s t-test). **b** Mean log_2_(fold change) of protein-coding genes divided into length deciles

Long non-coding RNAs (lncRNAs) have been found to show lower expression but higher tissue specificity than protein coding RNAs, with a high number of brain-specific lncRNAs [32–34]. There are five categories of genes encoding long non-coding RNAs and containing more than 50 expressed genes in our SKNBE samples: long intergenic non-coding RNA (lincRNA), antisense, processed_transcript, sense_intronic, sense_overlapping (Additional file 9: Table S6). Four of these biotypes, i.e. lincRNA, antisense, sense_intronic and sense_overlapping, are significantly upregulated with *p*-value < 0.001 (*p*-values are respectively 3.95*10^− 8^, 5.69*10^− 4^, 5.94*10^− 2^, 4.13*10^− 8^, 4.94*10^− 5^; one-sided Student’s t-test; Fig. 2a). In addition, we analyzed coding genes regarding a potential correlation between fold change and length. Analysis of the fold changes of coding genes binned into length deciles showed that gene length and fold change are positively correlated (R^2^ = 0.91, p-value = 1.78*10^− 5^; Fig. 2b). Collectively, these data suggest that *EDC3* knockdown preferentially affects the expression of long RNAs.

### Differentially expressed genes in *EDC3* knockdown cells are involved in synapses and cell cycle-related processes

**Fig. 3:**
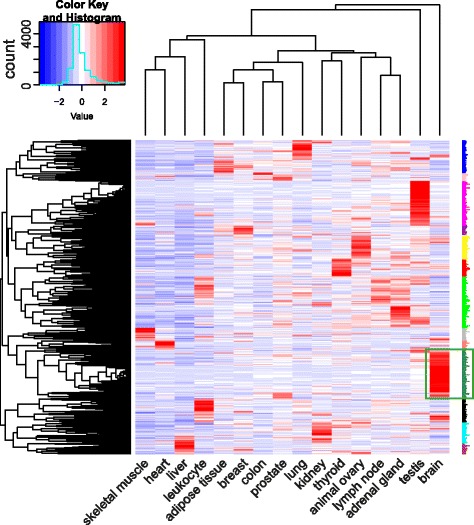
Tissue expression analysis of DEGs. Clustering of tissue expression profiles of DEGs retrieved by Expression Atlas. Tightly clustered genes, which are predominantly expressed in brain, are highlighted by the green rectangular box

In order to obtain insights into the functional consequences of EDC3 impairment and its potential effect on intellectual disability, we performed integrative bioinformatics analyses on the SKNBE transcriptome data. Out of the 16,321 expressed genes, 764 genes are differentially expressed in *EDC3* knockdown samples compared to controls (Benjamini adjusted p-value < 0.1) (Additional file 10: Table S7). In order to assess whether the DEGs are preferentially expressed in neuronal cells, we utilized available expression data in the Expression Atlas [25]. Clustering of the expression of 744 DEGs listed in the Expression Atlas revealed a cluster of 117 genes, which are highly expressed in brain but rarely in other tissues (Fig. 3; Additional file 11: Table S8). Pathway analysis of these 117 genes with DAVID revealed top enriched pathways related to synapses and coated vesicles (Additional file 12: Table S9).

**Fig. 4:**
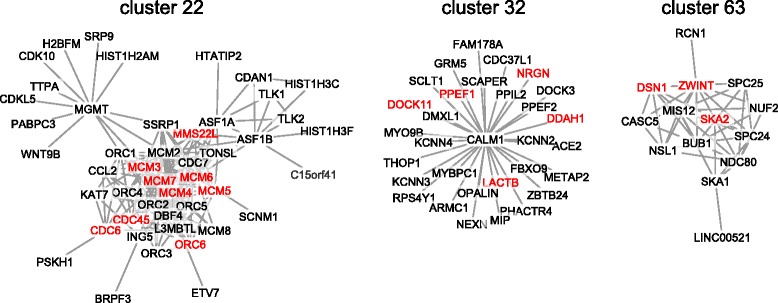
Clusters of protein-protein interaction network enriched in DEGs. Three clusters of the protein-protein interaction network published by Li et al. [27], defined in the original publication as clusters 22, 32, and 63, which are enriched in DEGs (red)

Functional enrichment analysis of all 764 DEGs performed with DAVID [29, 30] showed that the top enriched pathways are related to DNA replication and cell cycle (Additional file 13: Table S10). In order to investigate known protein-protein interactions between proteins encoded by the DEGs, we relied on a human protein-protein interaction network published by Li et al. [27] based on the extensive human protein interactome from BioGrid [28]. The authors had identified 817 clusters of densely connected proteins, with sparse interactions with proteins in other clusters. Out of the 817 clusters, 7 clusters (defined in the original publication as clusters 22, 32, 63, 217, 309, 339, and 383) were enriched in our DEGs (*p*-value < 0.05; hypergeometric test), with three of them (22, 32, and 63) containing at least 15 genes (Fig. 4). Functional annotation analysis with DAVID showed that cluster 22 was enriched for GO terms related to DNA replication and cell cycle, cluster 32 for terms related to ion channel activity, and cluster 63 for cell cycle and chromosome-related terms (Additional file 14: Table S11).

### Co-expression network analysis identifies a gene module enriched in synapses-related genes

**Fig. 5:**
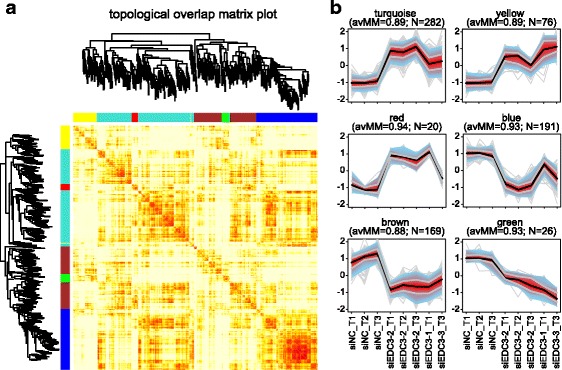
Modules identified by weighted co-expression network analysis on DEGs. **a** Topological overlap matrix (TOM) plot. Hierarchical clustering was applied to the TOM-based dissimilarity matrix to identify modules, i.e. groups of highly interconnected genes. The heatmap is a color-coded representation of the topological overlap between pairs of nodes. Each row/column corresponds to a gene and progressively darker red corresponds to higher topological overlap. The dendrogram and a color-coded module membership representation are shown to the left and above the heatmap. **b** Plots of expression profiles of genes in each of the six modules. Single gene profiles are shown in light grey, the black line corresponds to the median of module genes, the red area represents the interquartile range (25-75% of expression values) and the light blue area represents expression values of 90% of the data (5 - 95%). avMM: average module membership for genes in the module; N: number of genes in the module

In order to identify modules of co-expressed genes, we performed a weighted gene co-expression network analysis (WGCNA) [31, 35]. On the basis of the co-expression network learned on the 764 DEGs, it was possible to identify six modules of correlated genes, hereafter referred to as blue, turquoise, brown, yellow, red, and green module. All the modules are characterized by a high module membership score (0.91 on average). The high intra-module correlation appears evident also by visual inspection of the topological overlap matrix (TOM) plot, a color-coded depiction of the TOM-based dissimilarity values (Fig. 5a), and inspection of plots of the expression profiles in each cluster (Fig. 5b). For further investigations, we concentrated on the modules with the highest number of genes, i.e. blue, turquoise, and brown. Functional annotation analysis of these three modules revealed that the blue module was enriched in terms related to DNA replication and cell cycle, the turquoise module in terms related to synapses and coated vesicles, and the brown module in extracellular matrix (Additional file 15: Table S12). Furthermore, 56% of the 117 genes highly expressed in brain were assigned to the turquoise module (Additional file 11: Table S8; *p*-value = 6.33*10^-6^, hypergeometric test). Taken together, co-expression network analysis confirms the global association of DEGs with synapses-related processes and furthermore identifies a module of highly correlated synapses-specific genes.

## Discussion

In a previous study, we identified a homozygous variant in *EDC3* in two siblings with mild non-syndromic intellectual disability [12]. Molecular modelling suggested it to be a loss-of-function variant. Furthermore, whereas wild type EDC3 enhanced DCP2 decapping activity by two-fold in decapping in vitro assays, EDC3 with the identified variant failed to show any enhancement. In our transcriptome analysis from lymphoblastoid cell line samples of patients’ cells carrying the *EDC3* variant as well as from an in vitro EDC3 loss-of-function neuronal model based on siRNA knockdown we identified several hundred DEGs. The effect of EDC3 impairment at transcriptional level had not previously been studied and the large-scale transcriptional dysregulation we observed strengthens the role of EDC3 in mRNA decapping and degradation. Additionally, mRNAs containing AU-rich elements showed higher fold changes than mRNAs without AREs. EDC3 had been previously found to interact with TTP, an activator of the decay of ARE-containing RNAs [9]. The accumulation of ARE-containing mRNAs further supports the role of EDC3 in mRNA decapping and might contribute to the intellectual disability phenotype observed in our patients. Indeed, mRNA decapping has already been associated with ID, as variants in the scavenger decapping enzyme (*DCPS*), which is responsible for decapping in the 3′ to 5’ mRNA decay pathway, were identified in several unrelated patients who have intellectual disability [12, 36].

Transcriptome analyses in SKNBE neuroblastoma cells showed an upregulation of long non-coding and long coding RNAs. This indicates that EDC3 might be preferentially involved in decapping long RNAs. LncRNAs are involved in gene expression regulation and a large number of developmental processes, including neuronal development, and have been implicated in neuronal plasticity [33, 37–39]. A recent study has revealed a high co-expression between lncRNAs and known ID genes using genome-wide weighted gene co-expression network analysis [40]. Furthermore, dysregulated lncRNAs have been connected to a variety of neurodegenerative conditions such as Huntington’s disease, Alzheimer’s disease, autism spectrum disorders, and Angelman syndrome [41, 42]. The upregulation of lncRNAs in *EDC3* knockdown SKNBE cells might hint towards a potential causative correlation with the intellectual disability presented by our patients. However, the finding on lncRNAs should be interpreted with caution. Indeed, only a few have so far been functionally characterized [43] and more research about lncRNAs, their expression in different cell lines, and their role in neurological disease is needed. In addition to lncRNAs, long coding RNAs were also upregulated in EDC3 knockdown cells. In a study on *MECP2*, a gene leading to Rett syndrome (RTT) when disrupted, it was found that long genes were upregulated in *Mecp2* mouse mutant brain and human RTT brain [44]. A set of 466 long genes consistently dysregulated across multiple microarray datasets when MeCP2 function was perturbed contained many genes involved in neuronal modulation processes, axonal guidance, synaptic formation, or other neuronal functions [44]. The same study explored also a connection between the upregulation of long genes and fragile X syndrome, typically caused by inactivation of the fragile X mental retardation protein (FMRP). FMRP is involved in translation regulation, especially in inhibition of mRNA translation [45]. Gabel et al. showed that mRNAs targeted by FRMP were significantly longer than the genome average [44]. On the basis of our results and the above discussed previous findings, we hypothesize that the identified upregulation of long transcripts might contribute to the phenotype of intellectual disability in our patients. However, due to the current limited knowledge on the function of long RNAs, the mechanism underlying this contribution remains elusive.

Integrative analyses of SKNBE transcriptome data performed by different methodologies, including functional annotation analysis of DEGs and weighted co-expression network analysis, highlighted the potential involvement of two groups of pathways: synapses/coated vesicles on the one hand and DNA replication/cell cycle on the other. Synapses play a key role in the neuronal system and associations were found between intellectual disability and dysregulated genes known to be associated with synapses and synaptic vesicles traveling [46, 47]. Two well-known syndromes associated with intellectual disability, the Fragile X syndrome and Rett syndrome, affect synaptic function [48–53]. A study on the transcriptional regulator *NONO*, whose variants were identified in three patients with syndromic intellectual disability, found that one third of differentially expressed genes in transcriptome data from hippocampi of wild-type mice and mice with disrupted Nono were synaptosomal and connected for example to the regulation of dendritic spine morphology [54]. Furthermore, some non-syndromic intellectual disability cases are associated with impaired synaptic function. Recently, mutations of the gene *TRIO*, which is involved in neurite outgrowth and synaptic transmission, have been identified in four patients with mild to borderline intellectual disability and behavioral abnormalities [55]. The gene *IL1RAPL1* has been shown to be located in excitatory synapses, to play a role in synaptic differentiation, and be associated with cases of non-syndromic intellectual disability [56]. A recent study identified new variants, which seemingly decreased the synaptogenic activity of *IL1RAPL1* [56]. Further genes of the Rho GTPase family, like *OPHN1*, *ARHGEF6* and *PAK3,* have been implicated in non-syndromic intellectual disability and play a role in spine morphology and synaptic plasticity [57]. These examples strengthen the importance of synapses in the pathomechanism of intellectual disability. Taken together, the enrichment of DEGs in synapses-related pathways suggests a possible pathogenic mechanism of the identified variant in our patients. Whereas DNA replication and cell cycle are crucial for cell homeostasis and survival, their connection to the pathomechanism of neurodegenerative diseases is vague. It has been argued that cell cycle proteins are involved in DNA repair and neuronal plasticity in postmitotic neurons and show an abnormal expression in Alzheimer disease cells [58, 59]. Regarding our transcriptome data, it remains unclear whether the alterations in the expression of genes related to DNA replication and cell cycle contribute to the phenotype of intellectual disability in our patients. More research is necessary to investigate the role of DNA replication and cell cycle on neurodevelopment and adult neurons and possible connections to neurodegenerative diseases.

## Conclusions

Our analyses of transcriptional profiles from patients’ lymphoblastoid cell lines vs. those of healthy persons as well as from the neuroblastoma cell line SKNBE after *EDC3* knockdown vs. control strengthen the hypothesis of an involvement of EDC3 in mRNA degradation pathways and add further evidence supporting the pathogenicity of a previously identified *EDC3* variant in patients with mild non-syndromic intellectual disability. In addition, our results indicate an involvement of EDC3 in pathways related to synapses/coated vesicles and DNA replication/cell cycle and further suggest that long RNAs might be preferentially targeted by EDC3-mediated mRNA degradation. More research is needed to validate these findings on EDC3 function as well as increase our understanding of mRNA degradation pathways and their potential role in intellectual disability.

## Additional files

Additional file 1 **Table S1** *EDC3* siRNAs. Sequences of the three siRNAs targeting *EDC3* purchased from Invitrogen. (XLSX 8 kb)

Additional file 2 **Figure S1** Validation of SKNBE transcriptome data via qPCR. Comparison between fold changes obtained with RNA sequencing and with real time qPCR of selected genes. Direction of fold change was confirmed for 9 out of 10 assayed genes. (PDF 111 kb)

Additional file 3 **Table S2** Summary statistics for RNA-seq on patients’ lymphoblastoid cell line samples. Summary statistics for RNA sequencing of patient (P1, P2) and control (C1, C2) samples. (XLSX 8 kb)

Additional file 4 **Table S3** Results of differential expression analysis on patients’ lymphoblastoid cell line samples. Output of differential expression analysis performed with DESeq2 [23] for the 22,123 genes that passed independent filtering. HGNC symbols could be retrieved via biomaRt package for 17,975 genes. (XLS 3186 kb)

Additional file 5 **Table S4** Functional enrichment analysis of the DEGs in lymphoblastoid cell line samples. Functional annotation of the 235 DEGs was performed with DAVID [29, 30]. The table shows the top enriched terms (Benjamini-adjusted *p*-value < 0.1). (XLSX 9 kb)

Additional file 6 **Figure S2** Live Cell Images of differentiated and not differentiated SKNBE cells. a) Images after 72 h of culture. In cells treated with differentiation medium (from left to right: not transfected; treated with scrambled siRNA used as negative control [siNC]; treated with siEDC3-2) distinct neuronal elongations can be seen. Not transfected cells, which were cultured in normal proliferation medium (DMEM/HAM’s F12 + 10% FCS) without differentiation medium, maintained their more compact and round morphological shape. b) Images after 5 days of culture. All images were taken with Lumascope 500 (etaluma) 20× (Objective). (PDF 1458 kb)

Additional file 7 **Table S5** Summary statistics for RNA-seq on SKNBE samples. Summary statistics for RNA sequencing of knockdown samples with three different siRNAs targeting EDC3 (siEDC-1, siEDC3-2, siEDC-3) as well as control (siNC) samples relative to three replicate experiments (T1-T3). (XLSX 9 kb)

Additional file 8 **Figure S3** Principal component analysis plot for transcriptome profiles of SKNBE samples. Plot of the first two components obtained by principal component analysis of the five knockdown and three control samples. (PDF 309 kb)

Additional file 9 **Table S6** Number of expressed genes in SKNBE transcriptome data according to biotype. Overview of gene biotypes in Ensembl’s annotation file, number of expressed genes for each biotype, mean length, and length range. (XLSX 9 kb)

Additional file 10 **Table** **S7** Results of differential expression analysis on SKNBE samples. Output of differential expression analysis performed with DESeq2 [23] for the 16,321 genes that passed independent filtering. HGNC symbols could be retrieved via biomaRt package for 14,159 genes. (XLS 2378 kb)

Additional file 11 **Table S8** DEGs predominantly expressed in brain. List of 117 DEGs that are predominantly expressed in brain according to Expression Atlas. The last column shows the gene’s module membership in WGCNA analysis. (XLSX 11 kb)

Additional file 12 **Table S9** Functional enrichment analysis of DEGs predominantly expressed in brain. Functional annotation of the 117 DEGs predominantly expressed in brain was performed with DAVID. The table shows the top enriched terms (Benjamini-adjusted *p*-value < 0.1). (XLSX 9 kb)

Additional file 13 **Table S10** Functional enrichment analysis of the SKNBE DEGs. Functional annotation of the 764 DEGs was performed with DAVID. The table shows the top enriched terms (Benjamini-adjusted p-value < 0.1). (XLSX 10 kb)

Additional file 14 **Table S11** Functional enrichment analysis of protein-protein interaction clusters enriched in DEGs. Functional annotation of the protein clusters 22 (50 genes), 32 (31 genes), and 63 (15 genes) enriched in DEGs was performed with DAVID. The table reports the top 15 ranked (by Benjamini-adjusted p-value) pathways for each cluster. (XLSX 11 kb)

Additional file 15 **Table S12** Functional enrichment analysis of the modules identified by WGCNA. Functional annotation of the modules was performed with DAVID. The table reports the top 15 ranked pathways (by Benjamini adjusted p-value) relative to the genes in the blue module (*N* = 191), the turquoise module (*N* = 282), and the brown module (*N* = 169). (XLSX 11 kb)
