# Supplementary figures and images for: Integrative bioinformatics analysis characterizing the role of EDC3 in mRNA decay and its association to intellectual disability
Source: BMC Med Genomics. 2018 Apr 23;11:41. doi: 10.1186/s12920-018-0358-6 (PMC5914069; doi:10.1186/s12920-018-0358-6)

Scheller U *et al.*, Supplementary Figure S1

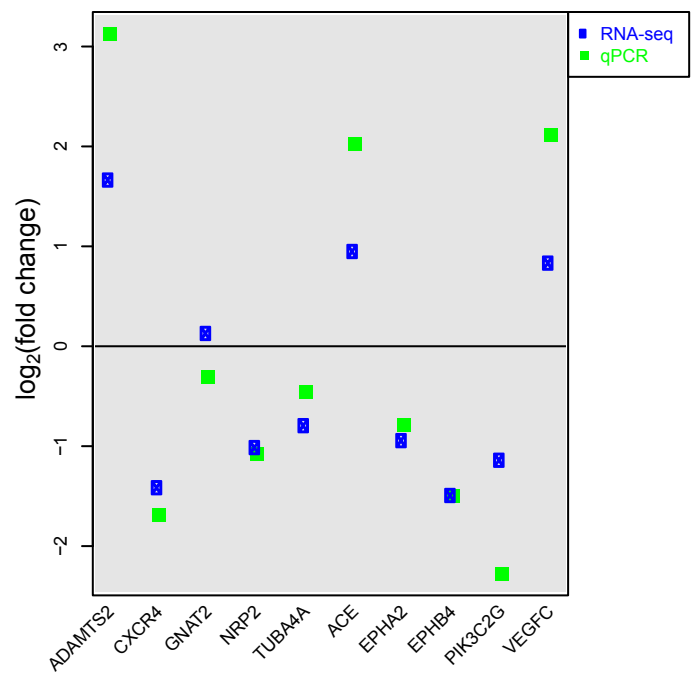

Supplement: Supplementary file 2 — Figure S1 Validation of SKNBE transcriptome data via qPCR. Comparison between fold changes obtained with RNA sequencing and with real time qPCR of selected genes. Direction of fold change was confirmed for 9 out of 10 assayed genes. (PDF 111 kb) [file 12920_2018_358_MOESM2_ESM.pdf]

Scheller U *et al.*, Supplementary Figure S2

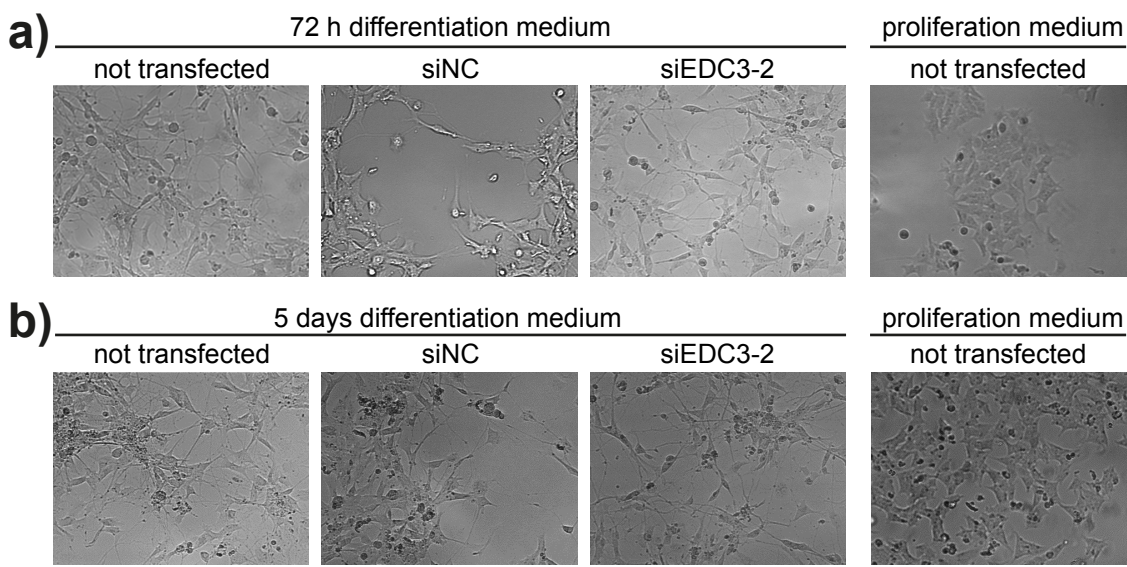

Supplement: Supplementary file 6 — Figure S2 Live Cell Images of differentiated and not differentiated SKNBE cells. a) Images after 72 h of culture. In cells treated with differentiation medium (from left to right: not transfected; treated with scrambled siRNA used as negative control [siNC]; treated with siEDC3-2) distinct neuronal elongations can be seen. Not transfected cells, which were cultured in normal proliferation medium (DMEM/HAM’s F12 + 10% FCS) without differentiation medium, maintained their more compact and round morphological shape. b) Images after 5 days of culture. All images were taken with Lumascope 500 (etaluma) 20× (Objective). (PDF 1458 kb) [file 12920_2018_358_MOESM6_ESM.pdf]

# Scheller U *et al.*, Supplementary Figure S3

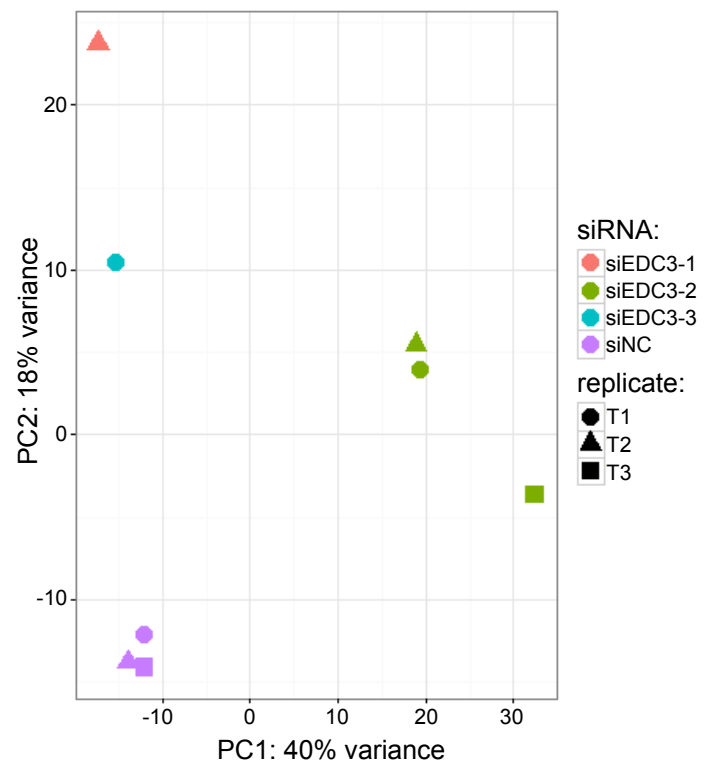

Supplement: Supplementary file 8 — Figure S3 Principal component analysis plot for transcriptome profiles of SKNBE samples. Plot of the first two components obtained by principal component analysis of the five knockdown and three control samples. (PDF 309 kb) [file 12920_2018_358_MOESM8_ESM.pdf]
